# Avatar led interventions in the Metaverse reveal that interpersonal effectiveness can be measured, predicted, and improved

**DOI:** 10.1038/s41598-022-26326-4

**Published:** 2022-12-19

**Authors:** Arjun Nagendran, Scott Compton, William C. Follette, Artem Golenchenko, Anna Compton, Jonathan Grizou

**Affiliations:** 1 Mursion Inc., San Francisco, CA USA; 2 Psychiatry and Behavioral Sciences, Duke University, Durham, NC USA; 3 Department of Psychology, College of Science, University of Nevada, Reno, NV USA; 4 School of Computing Science, University of Glasgow, Glasgow, UK

**Keywords:** Human behaviour, Computational science, Software

## Abstract

Experiential learning has been known to be an engaging and effective modality for personal and professional development. The Metaverse provides ample opportunities for the creation of environments in which such experiential learning can occur. In this work, we introduce a novel interpersonal effectiveness improvement framework (ELAINE) that combines Artificial Intelligence and Virtual Reality to create a highly immersive and efficient learning experience using avatars. We present findings from a study that uses this framework to measure and improve the interpersonal effectiveness of individuals interacting with an avatar. Results reveal that individuals with deficits in their interpersonal effectiveness show a significant improvement (*p* < 0.02) after multiple interactions with an avatar. The results also reveal that individuals interact naturally with avatars within this framework, and exhibit similar behavioral traits as they would in the real world. We use this as a basis to analyze the underlying audio and video data streams of individuals during these interactions. We extract relevant features from these data and present a machine-learning based approach to predict interpersonal effectiveness during human-avatar conversation. We conclude by discussing the implications of these findings to build beneficial applications for the real world.

## Introduction

Over the last decade, advances in computational hardware have accelerated the adoption of virtual reality and artificial intelligence technologies across the workplace. This wave of adoption has led to social media platforms and companies embracing the term “Metaverse,” referring to an aspirational seamless boundary between the physical realm and computer-generated realms (i.e., an integrated network of persistent, online virtual, or augmented environments^1^. Technological advancements in virtual and augmented reality technology, artificial intelligence, high-speed global connectivity, and rendering devices with powerful onboard computing have resulted in the creation of virtual worlds (computer-generated realms in the Metaverse) within which experiential learning can occur. With the Metaverse hoping to encompass many aspects of our current and future existence, with the aim of enhancing our connectivity and the quality of our shared experiences, the obvious question is how each of us individually interacts with and manifests across the real and virtual worlds, which leads us to “avatars”, a concept extensively studied by the research and scientific community. The term “avatar” is deep-rooted in hindu mythology and was used to describe the descent of a divine entity from celestial realms to terrestrial regions^2^, often taking a form that was optimized to complete a specific objective. A virtual avatar is described as a perceptible digital representation whose behaviors reflect those executed, typically in real time, by a specific human being^3^. Individuals who can influence or directly control their virtual counterparts (avatars) are referred to as inhabiters^4^. Previous research has shown that avatars can convincingly simulate social scenarios and adaptively steer conversations^5,6^, while eliciting strong levels of social influence^7^^,^^8^^,^^9^. Avatars are also able to elicit similar emotional responses in a conversational partner when compared to interacting with a live human^10^. These findings show promise for training in industries such as customer service, corporate leadership, sales, and healthcare, with the primary goal of enabling professionals to deal with challenging situations they may encounter in the real world. Research has shown that training in such virtual worlds alleviate high costs associated with making mistakes in the real world^11^^,^^12^.

More recently, the use of personalized 3D avatars in a 2D video conferencing context has indicated a higher level of perceived social presence by participants when compared to traditional video^13^. Using avatars has many advantages when compared to traditional role-play based training programs^14^ (a comprehensive review can be found in Van Ments^15^, Lane and Rollnick^16^. The use of virtual environments for learning is already being implemented in many professional training programs, particularly to alleviate high costs associated with making mistakes in the real world^11,12^. Such virtual environments are easily scalable and customizable (e.g., outdoors, offices, public spaces, conference rooms, etc.). Providing the ability for individuals to enter and exit virtual environments seamlessly, either as themselves, or in the form of avatars, further amplifies the effectiveness of the training since the avatar manifestation (i.e., age, race, demographic, personality etc.) adds a layer of complexity that can present new learning challenges^17,18,19^.

Despite the large body of research centered around human-avatar interaction in virtual worlds, there are currently no available frameworks, to our knowledge, that can measure the effectiveness and the outcome of interpersonal interactions between avatars and humans.

Exactly what constitutes the construct of interpersonal effectiveness is not universally agreed upon (Phillips et al., 2016), but empirical data suggests that humans, in general, are social and predisposed to form and maintain close interpersonal relationships^20^^,^^21^^,^^22^. An important factor that goes into forming and maintaining interpersonal relationships is the ability to recognize the impact one is having on another person with a view to achieving a social goal. This ability, referred to as interpersonal effectiveness, is closely associated with conventional definitions of Emotional Intelligence^23^ or Self-Awareness. The term emotional intelligence (EI) was first defined by Salovey and Mayer^24^ and described as the “ability to perceive accurately, appraise and express emotions, the ability to understand and regulate emotions and to use this information to guide one’s thinking and actions.” Work by Miao, Humphrey and Qian^25^ has suggested that there is a positive association between EI and authentic leadership, which has practical implications for the workplace as it allows leaders to achieve desirable outcomes across organizational levels. Further, work by Gardner et al.^26^ found that leaders who score high on the emotion perception branch of EI are better at perceiving others’ emotions, allowing them to create empathetic bounds and be a more authentic leader. Similarly, leaders who are aware of their own emotions and understand the impact they have on others (e.g., self-awareness) are perceived by their subordinates as being more effective leaders^21^. These findings suggest that it is particularly important to be able to recognize one’s interpersonal effectiveness when working in both personal and professional settings and leadership roles. We also believe that interpersonally effective behavior is learned. Such behaviors are selected based on the consequence they have for the person emitting them. Behaviors that increase the likelihood of achieving a goal are strengthened.

While the importance of interpersonal effectiveness in achieving both personal and business outcomes is well established, there are no defining frameworks to help quantify this as a measure. We propose such a framework, called ELAINE (**E**xperiential **L**earning using **A**vatars to improve **IN**terpersonal **E**ffectiveness), by immersing individuals into a virtual world where they interact with an avatar across four interpersonally challenging conversations (referred to here as Scenarios). Virtual Reality and Artificial Intelligence were used to create highly realistic avatars, inhabited by a single individual who interacted with all participants during this study. The avatar control interface allowed the inhabiter to continuously assess the interpersonal effectiveness of the participant interacting with the avatar during the interaction. In addition, a simple survey was administered at the end of the interaction to assess whether the participant was successful in achieving the goal of the challenging conversation. A detailed description of the study design is included in the Methods section. In the next section we present results from analyzing over 200 such interactions.

The primary objectives of the study were: (1) to evaluate the association between continuous ratings of interpersonal effectiveness collected during conversations (referred to as IMPACT) and post conversation ratings of success (referred to as SURVEY); (2) to evaluate improvement in ratings of interpersonal effectiveness (IMPACT, SURVEY) between Scenario 1 (screening conversation) and Scenario 4 (post-assessment conversation); (3) to explore whether improvement in ratings of interpersonal effectiveness (IMPACT, SURVEY) differ between participants rated as “successful” or “unsuccessful” following the initial screening conversation (Scenario 1); and (4) to evaluate whether video and audio data streams collected during conversations can be used to predict post-conversation success probability (SURVEY scores). We hypothesized that (1) continuous ratings of interpersonal effectiveness (IMPACT) and post-conversation ratings of success (SURVEY) will be positively correlated; (2) participants' interpersonal effectiveness scores (IMPACT) will significantly improve between Scenario 1 and Scenario 4 and those participants rated as “unsuccessful” at the end of Scenario 1 will show more improvement than those rated as “successful” by Scenario 4. We also hypothesize that verbal and nonverbal behaviors of participants during a Scenario will predict interpersonal effectiveness. This stems from evidence in the literature that survey-based measures of emotional intelligence and hence, interpersonal effectiveness, may be less valid, and that data-based measures such as kinesics and prosodics may yield better results^27^.

## Methods

### Study design

To evaluate our hypotheses, participants completed 4 different conversations with the same avatar in a single session: a screening conversation (Scenario 1), 2 training conversations (Scenarios 2 and 3), and one assessment conversation (Scenario 4). The four Scenarios used for the study can be found here (https://web.mursion.com/references/StudyScenarios.pdf). The order of the training conversations (Scenarios 2 and 3) was randomly assigned to minimize the possibility of order effects. Participant improvement in interpersonal effectiveness was assessed by evaluating change in SURVEY and IMPACT using a pretest–posttest design (between Scenarios 1 and 4). These are defined below:

### Primary outcomes

#### Post-conversation survey rating of success (SURVEY)

Prior to each conversation, participants read a brief description of the Scenario for backstory and context. The description also presented the participant with a pre-specified outcome (i.e., goal) to be achieved by the end of the conversation. Participants were allowed to use any strategy of their choosing. Following each conversation, the inhabiter and the participant were asked to indicate how likely this outcome was achieved using the SURVEY-I or SURVEY-P, respectively. For the inhabiter, the SURVEY-I consisted of the average of the following two items, each rated on a 1-to-10 Likert-scale:

1. Following this conversation, indicate how likely you are to try < insert outcome > , where 1 = “Extremely Unlikely” and 10 = “Extremely Likely.” (SURVEY-I1)
2. In coming to a decision, did you feel your views were considered or dismissed, where 1 = “Extremely Dismissed” and 10 = “Extremely Considered.” (SURVEY-I2)

For participants, the SURVEY-P consisted of a single item rated on 1-to-10 Likert-scale:

1. Based on the conversation you just had, indicate how likely < Name of avatar > will try < insert outcome > , where 1 = “Extremely Unlikely” and 10 = “Extremely Likely.”

The SURVEY-I consisted of the average of two items, rather than a single item, to correct for the possibility that a participant may use coercion as a strategy to achieve the goal (e.g., “you must do < insert goal > because I’m your superior and I’m telling you to do so!”). This data was also recorded as a binary indicator of conversation outcome (success/failure) with SURVEY scores ≥ 7 indicating a “successful” conversation and scores < 7 indicating an “unsuccessful” conversation.

#### Continuous rating of interpersonal effectiveness (IMPACT)

This measure captured the continuous impact the learner was having on the inhabiter (and consequently, the avatar) by taking into account the behavior of the learner, both verbal and non-verbal, during the conversation with respect to the pre-specified outcome of that Scenario. During each conversation, the inhabiter provided a continuous rating of the performance of each participant using a three-level ordinal scale (positive, neutral, negative) captured via the mechanics described in Sect. 3.2.1. This process resulted in a continuous stream of data that reflected the participant’s performance throughout the conversation from the perspective of the inhabiter. This data was then processed to generate an overall IMPACT score, with higher values reflecting better interpersonal effectiveness. Following the screening conversation (Scenario 1), participants were classified as “successful” or “unsuccessful” with respect to achieving the pre-specified outcome. Participants whose Scenario 1 SURVEY scores were ≥ 7 were defined as “Successful” and those with scores < 7 were defined as “Unsuccessful.” Following each conversation, participants filled out the SURVEY questionnaire. The inhabiter completed the SURVEY-I and the participant completed the SURVEY-P. At the end of the final assessment conversation (Scenario 4), participants were also asked to fill out a post-participation questionnaire. A link to all the questionnaires used in the study can be found here (https://web.mursion.com/references/AllSurveys.pdf).

### Artificial intelligence and virtual reality framework

**Figure 1:**
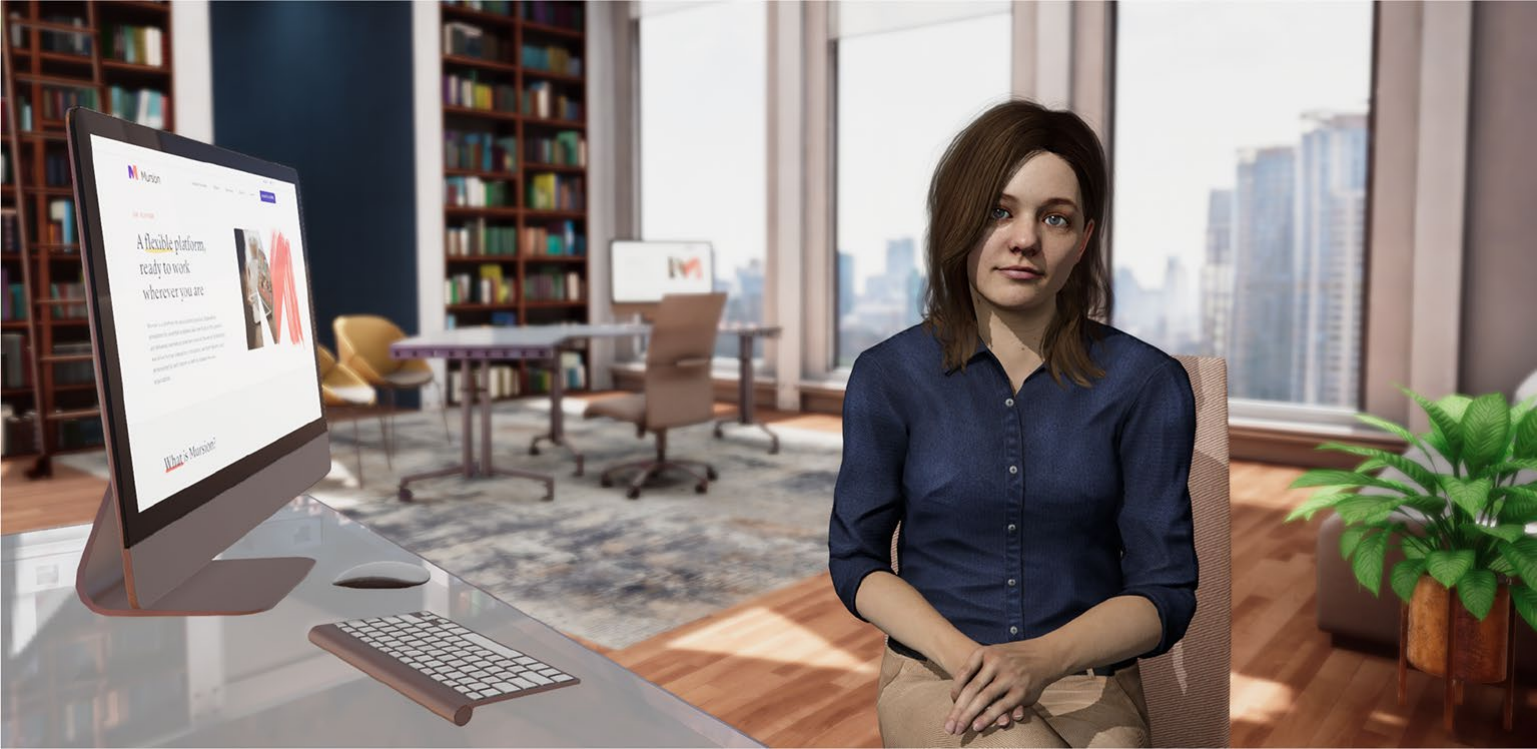
The immersive virtual world used to simulate a challenging conversation with a colleague, represented by an avatar in a workplace setting.

The VR software used for the study was built using the game development engine Unity^28^. Photogrammetry was used to generate the avatars. The virtual environments used were modeled in Maya^29^ and imported into the rendering engine. A screenshot of this software is shown in Fig. 1. The software had two synchronized peer to peer networked components,an authoritative component that was used by an inhabiter to control the avatar and a non-authoritative component that was used by participants to interact with the avatar. The authoritative instance was controlled by an inhabiter and responsible for creating a secure networked room using an adapted native version of the Web Real Time Communication protocol (WebRTC^30,31^) . The real time audio input from an inhabiter was represented as mel-spectrograms at 24 kHz sampling rate featuring frequencies from 10 Hz to 11.66 kHz distributed in 93 mel bands. A speaker-independent autoencoder trained in an unsupervised manner to predict phonetic content from the audio data.

To improve the stability of the results, a moving average filter was first applied over the predicted viseme probabilities followed by an uncertainty threshold for transitions. This data was used to synchronize the avatar’s lips with the inhabiter’s speech. For avatar non-verbals, we recorded and studied a library of videos of individuals interacting with each other in a video-based conferencing system and in recreated natural settings, paying particular attention to the facial expressions, microgestures, body poses and head motion of these individuals.

We extracted facial features using the OpenFace library^32^ and audio features using the Praat audio library^33^ for frontal-facing videos in our library. We established correlations between the length and signal characteristics of audio segments and non-verbal behaviors such as facial expressions, body pose, frequency of body pose changes, head motion, and frequency of microgestures among other things during these interactions. We analyzed these correlations when an individual was speaking separately from when an individual was listening during the interaction. This data was used to inform the creation of an animation library using a combination of Optitrack/Motive, Xsens Suit, Manus gloves, and a head mounted GoPro camera system, with nearly 40 min of animation. Maya and MotionBuilder were used to create the avatar control rig. Using the previously described correlations between audio segments and nonverbal behaviors, we used a real-time decision tree framework^34,35^ to drive the facial expressions, posture changes, gestures, and microgestures of the avatar. A brief video demo of the framework used to drive an avatar using purely audio signals can be viewed online at the following URL here (https://web.mursion.com/references/AudioBasedAI.mp4).

### Rating interface

The inhabiter (real person) was given a keyboard interface and trained via a certification process (see appendix) to use the keyboard to assess the moment-by-moment interpersonal performance of the participant during the interaction. The interface allowed for the selection of three impact states: positive, neutral, or negative. If the inhabiter thought that the performance of the participant was positive at any given moment, he or she would indicate this rating using the keyboard. This rating would continue as positive until the inhabiter felt that the performance of the participant was either neutral or negative, at which point they would indicate a change in their moment-by-moment rating (see IMPACT, Continuous Rating of Interpersonal Performance). At the end of the conversation, the inhabiter also rated whether the participant achieved the outcome of the interaction using the SURVEY (see Post-conversation Rating of Success) launched via the software interface. Throughout the interaction, the inhabiter was able to receive a video and audio feed of participants. In addition to the continuous impact assessments, the inhabiter was also trained to indicate any events of interest in the conversation with a positive or negative valence. These were separately time stamped and labeled as 4 and 5 respectively using the keyboard interface (page up and page down keys). The inhabiter was allowed to make these valence changes and mark points of interest as often as needed throughout the interaction, based on the performance of the participant. It was important to close the loop between the continuous impact data and events of interest indicated by the inhabiter and the behavioral manifestation of the avatars. This data was therefore used as an input to the decision tree algorithm, providing us the ability to alter decision nodes during traversal. This ensured that the body language and facial expressions of the avatar reflected the valence (positive, negative, or neutral) that the participant was having in the moment. Additionally, the events of interest triggered subtle changes in facial expressions or microgestures that were aligned with the valence of the event, providing immediate contingent feedback to the participant about the interaction.

### Sample characteristics

100 individuals were recruited for this study via YouGov’s online panel^36^. Out of the 100 recruited participants, 75 completed the baseline survey. Some participants (~ 6%) experienced networking or technical issues during the interactions. Additionally, there were instances where the participants did not complete the interaction-specific questionnaires (~ 26%). Our final data set used in the analysis therefore contains 204 interactions from 51 participants (4 complete uninterrupted interactions and associated survey data for each participant). Standard ethical protocols were followed for information, informed consent and recording consents for all participants and administered by YouGov (https://yougov.co.uk/about/). All methods were carried out in accordance with relevant guidelines and regulations and experimental protocols were approved by YouGov. Of the N = 51 valid participants, 59% were female and 41% were male. 92% were employed full time, 8% were self-employed, and the average age was 41.2 years (SD = 9.2). The study sample closely mirrored the local population with respect to race and ethnicity based on recent US census data, with participants self-identifying as 84% White, 6% American Indian, 2% Black, 4% Asian, and 4% Latinx. With respect to self-reported total annual income, 14% were between $25,000 and $49,999, 34% were between $50,000 and $99,999, and 52% were $100,000 or more.

### Study setting

The study was conducted at a physical location in Portland, OR where standardized hardware was set up in a small, closed room where the participants sat at a table with a laptop. This was meant to simulate an office environment for individuals to experience the simulations privately. Lighting and all other stimulus conditions were held constant for all participants through all the simulations. A moderator facilitated the study and was available to answer any questions that participants had.

### Statistical analysis

Participants with complete data at each conversation were included in the analyses (N = 51). All statistical tests were evaluated using SAS Statistical Software, version 9.4 TS Level 1M7 (SAS Institute, Cary, NC). A series of simple Pearson’s correlations were conducted via PROC CORR to examine the relationship between IMPACT and SURVEY scores within each scenario. A repeated measures ANOVA was then conducted to examine change in IMPACT scores across between baseline screening (Scenario 1) and post-assessment (Scenario 4). The main independent variables of interest were group (“successful” versus “unsuccessful”) and measurement time (4 scenarios) and the group by time interaction. An unstructured correlation structure was used to capture the within-person correlation over time. The regression model was implemented using PROC MIXED and the Ken-Warl-Roger option was used to obtain the correct denominator degrees of freedom for the F-tests. Residual error terms were assumed to follow a mean-0, normal distribution. The fitted model was used to report average IMPACT scores within each level of the independent variables and to make inferences about within-in and between-group differences across the scenarios. All tests were 2-sided and *p* < 0.05 was considered statistically significant. The adaptive step-down Bonferroni adjustment (as implemented in PROC MULTTEST) was used to control the overall (family-wise) error rate of all unplanned comparisons. Finally, PROC FREQ was used to conduct a 2 × 2 Chi-Square Goodness of Fit Test to determine whether the proportion of participants rated as “successful” versus “unsuccessful” changed significantly between Scenario 1 and 4.

### Automated analysis

**Figure 2:**
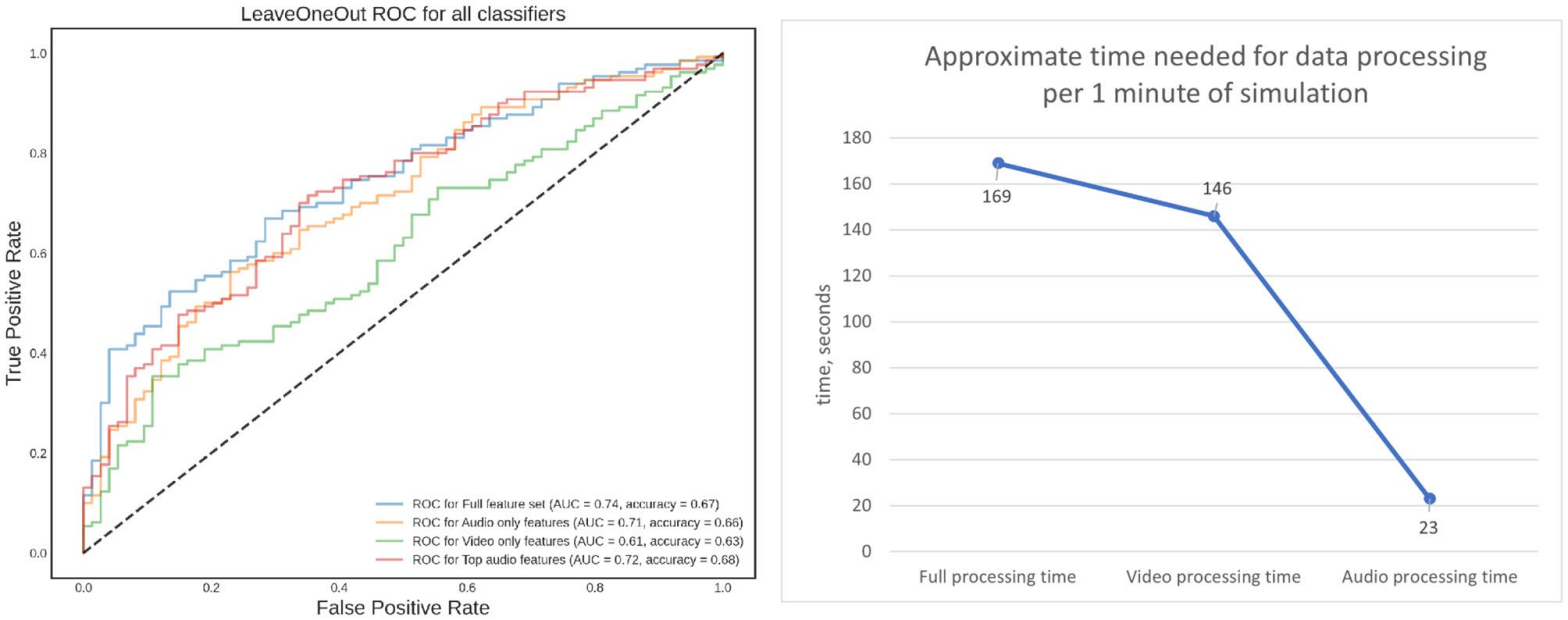
Left: ROC curves for the SVM trained on full feature set, audio only, video only, and top selected features. Right: Approximate time needed for data processing per one minute of simulation, in seconds. By removing video features, computing time was reduced by 85%.

The interactions were analyzed to discover any correlations between the underlying audio and video streams and the final outcome of the interaction between the participants and the avatar. The top-20 features were selected based on their importance (coefficient weights) assigned by a Support Vector Machine classifier. A correlation matrix of these top-20 features was then computed, following which the number of features was reduced from 53 to 17 using thresholding. This ensured that highly correlated features that may be redundant were omitted. To check that classifiers don’t overfit, we performed Leave-One-Out cross-validation, where each sample is used once as a test set, and all the remaining samples are used as a training set. For the video streams recorded, a ‘video feature set’ was created using pre-trained machine learning models, specifically, OpenFace (OF)^32^ and an emotion recognition model^37^. First, a participant’s video was processed with OF: for each frame of video extracted, the face was detected and cropped,then, an enhanced pretrained model for emotion recognition^38^ detected a set of 48 emotions for each frame. Based on the work by Ahn^39^ (see Fig. 2. Two dimensional circumplex space model and its emotional sample), these 48 emotions were mapped onto a 2D space (or 2D map), with each emotion characterized as either active or passive, and either positive or negative. Coordinates for each emotion were interpreted from the 2D space, and emotions were grouped into 8 clusters: active-positive, active-negative, passive-positive, strongest-passive-positive (background emotions like ‘calmness’ and ‘concentration’), passive-negative, and three other clusters for the perceived engagement of a participant. For each frame of video an emotion vector was calculated as

$$\upnu ={\sum }_{\mathrm{i}}{\mathrm{probability}}_{\mathrm{i}}\cdot {\mathrm{coordinates}}_{\mathrm{i}}$$

**Figure 3:**
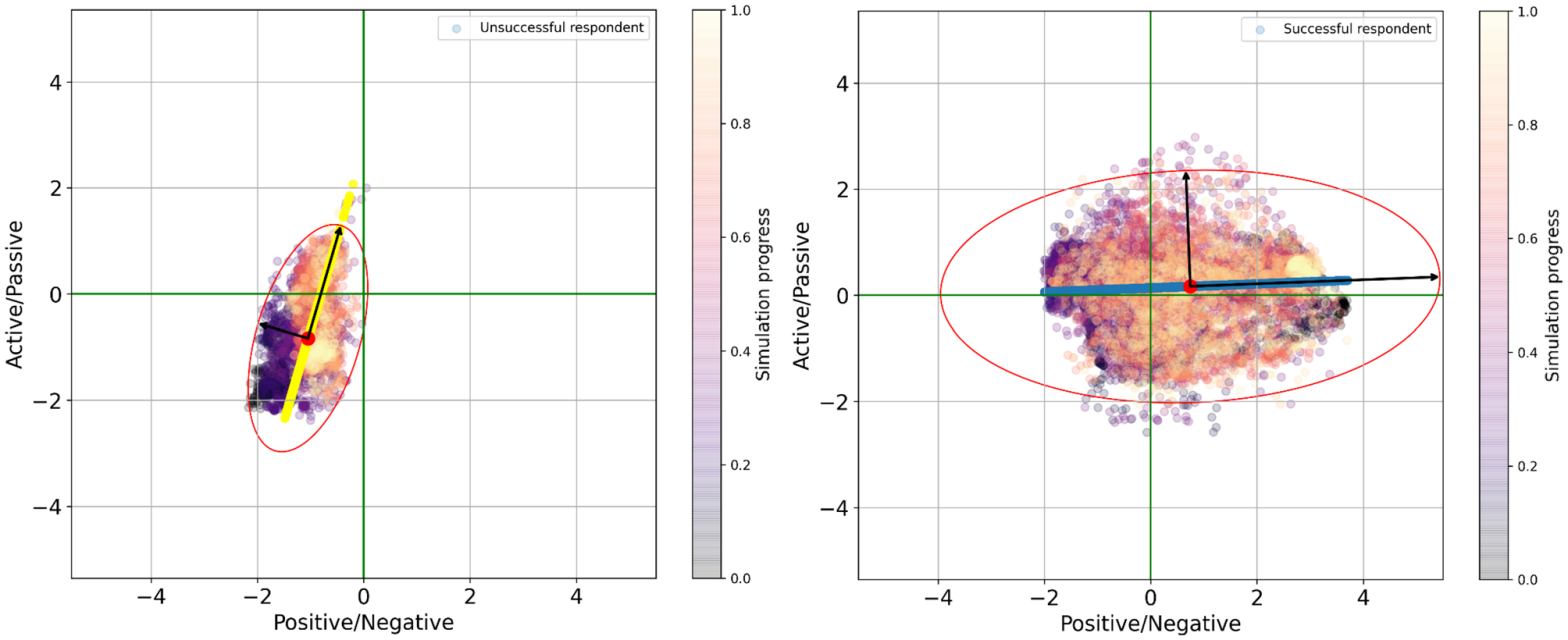
(Left) Low Interpersonal Effectiveness: Probabilities of extracted facial features perceived by an emotion detection algorithm are concentrated in the negative region, and mostly in the passive quadrant. The center of mass is located in the passive-negative region. (Right) High Interpersonal Effectiveness: The probabilities of extracted facial features perceived by an emotion detection algorithm for the entire interaction are well-distributed over the quadrants, with their center of mass located in the active-positive region. Also shown in both images are the confidence ellipsis, centers-of-mass, main components of Principal Component Analysis (PCA) decomposition, and PCA-explained-variance-vectors. Since these features were extracted for a video sequence, time (normalized) is indicated as color and accounts for each frame of processed video. The color therefore indicates the change in detected emotional expression for a participant during a simulation. For example, for the individual with a low interpersonal effectiveness score (Left), we can see that at the start of the interaction, perceived emotions were mostly negative (dark blue cluster centered around [− 1.7, − 1.7], but by the end of the simulation, their perceived emotions became slightly **more** positive (yellow cluster centered at [0.8; − 1.2]). (Generated with Google Colab).

A comparison of the clusters belonging to two representative subjects (successful and unsuccessful) revealed a significant disparity in the footprint of the emotion vectors (see Fig. 3). This provided the basis to train a classifier based on the features obtained by the emotion recognizer. Principal Component Analysis (PCA) was done based on the emotion vectors, and the center-of-mass was calculated for each session. The second set of features, an ‘audio feature set’, was taken from the participants’ audio with a version of the Praat software^40^. Each audio file was processed to reduce noise, and silences in the audio segments were removed.

Three subsets of features were then extracted from each audio segment. The first subset consists of basic audio statistics, like mean, median, minimum, and maximum values of the fundamental frequency F0^33^, jitter (variation in periods), shimmer (cycle-to-cycle variation in intensity), harmonicity (degree of acoustic periodicity, also called Harmonics-to-Noise Ratio (HNR)), mean and median values of the first four formants (frequency peaks in the spectrum which have a high degree of energy, especially prominent in vowels,each formant corresponds to a resonance in the vocal tract). All these features were calculated using a moving window of 0.5 s and no overlap, with the final feature set having a mean value calculated across all these windowed segments. The second subset included the average formant, formant dispersion, interaction of glottal-pulse rate and vocal-tract length in judgements of speaker size, sex, and age, vocal tract length, and formant spacing. The third subset consisted of an overall snapshot of the interaction including duration of speech, number of syllables, phonation time, number of pauses, speech rate (number of syllables divided by speech duration), and articulation rate (number of syllables divided by phonation time).

## Results

Our final dataset^41^ consisted of 51 individuals who each completed all four challenging conversations for a total of 204 interactions. Analysis of this data was performed to assess if an individuals’ interpersonal effectiveness could be measured, predicted, and improved as a result of interactions with an avatar:

### Is there a relationship between continuous ratings of interpersonal effectiveness (IMPACT) and post-conversation ratings of success (SURVEY)?

To evaluate the relationship between IMPACT and SURVEY ratings, separate Pearson’s correlations were conducted between these two measures at each scenario. The correlation was 0.68 (*p* < 0.0001) for Scenario 1, 0.13 (*p* = 0.3435), 0.67 (*p* < 0.0001) for Scenario 3, and 0.51 (*p* < 0.0001) for Scenario 4. With the exception of Scenario 2, these findings suggest that those participants who were rated as more likely to achieve the Scenario goal (SURVEY) were also more likely to be interpersonally effective (IMPACT) during the conversation.

### The relationship between continuous ratings of interpersonal performance (IMPACT), practice, and post-conversation rating of success (SURVEY)

**Figure 4:**
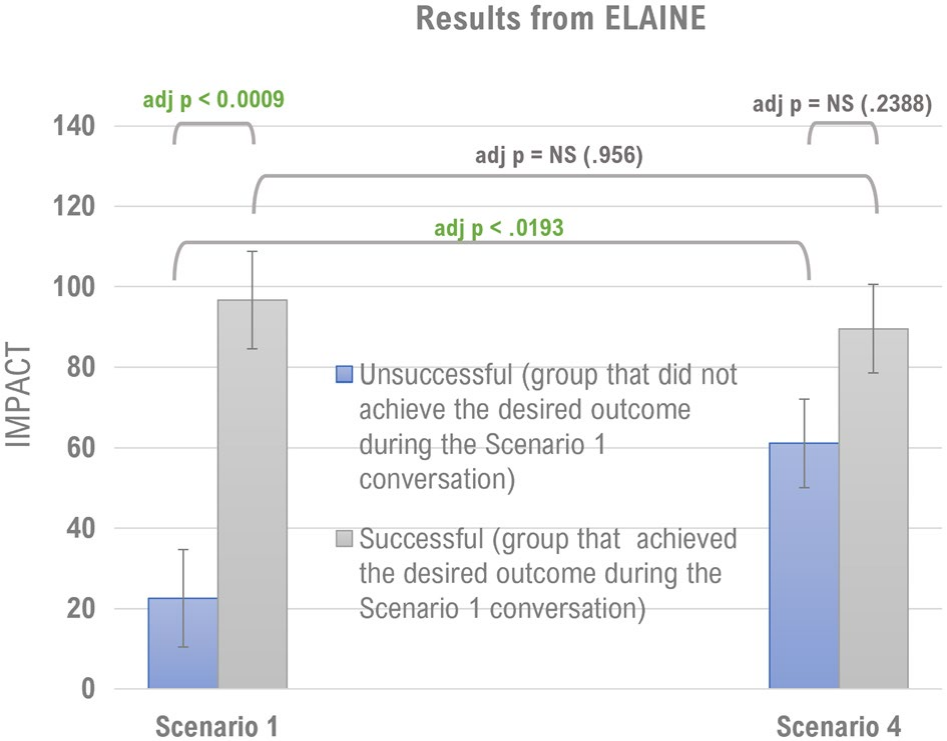
(Left): Individuals who were identified as having deficits after the baseline Scenario (Unsuccessful Group) showed a significant improvement by Scenario 4. A between group comparison reveals that the unsuccessful group after Scenario 1 showed no significant differences from the successful group after Scenario 4. The difference in interpersonal effectiveness for the Successful group between Scenario 1 and Scenario 4 was not significant.

Results from the repeated measures ANOVA reveal a significant main effect for group (SUCCESS), F (1, 49) = 14.26, *p* < 0.0004 and a significant time X group interaction (SUCCESS X SCENARIO), F (1, 49) = 5.86, *p* < 0.0193. The main effect for time (SCENARIO) was not statistically significant (*p* = 0.1029). The significant main effect for the group (SUCCESS) suggests that the average IMPACT scores across conversations was significantly different between “successful” and “unsuccessful” participants. The predicted average mean score for “successful” participants was 93.11 (SE = 10.05) and 41.86 (SE = 9.11) for “unsuccessful” participants: a difference of 51.25 (SE = 13.57) points. However, the significant interaction between group and time (SUCCESS X SCENARIO) suggests that the average within-in group change between conversation 1 and 4 varied by success status. Planned pairwise comparisons between those rated as “successful” versus “unsuccessful” at Scenario 1 revealed that successful participants had significantly higher average IMPACT scores (M = 96.70, SE = 13.38) compared to unsuccessful (M = 22.55, SE = 12.13), **adj p < 0.0009**. Additional planned pairwise comparisons between Scenario 1 and 4 within each level of success status revealed that “unsuccessful” participants had significantly higher average IMPACT scores at Scenario 4 compared to Scenario 1 (Scenario 1 M = 22.55, SE = 12.13 and Scenario 4 M = 61.18, SE = 9.99), with an average IMPACT score difference of 38.63 (SE = 12.71), **adj p < 0.0193**. Although participants rated as “successful” at Scenario 1 had lower average IMPACT scores at Scenario 4 (Scenario 1 M = 96.70, SE = 13.38 and Scenario 4 M = 89.52, SE = 11.01), this average difference in IMPACT scores of 7.17 (SE = 14.03) was not statistically significant (**adj p = 0.9560**). Figure 4 presents the average IMPACT scores along with their standard errors at each time point by success status and relevant p-values for each pairwise comparison between the screening conversation (Scenario 1) and the assessment conversation (Scenario 4). Between-group comparisons at each scenario found that at Scenario 1 “unsuccessful” participants had significantly lower IMPACT scores on average than “successful” participants, and by Scenario 4 this difference was not statistically significant (**adj p = 0.2388**).

### Does the proportion of those rated as successful change from Scenario 1 to Scenario 4?

Following the baseline screening, 23 (45.1%) of participants were rated as “successful” and 28 (54.9%) were rated as “unsuccessful.” By the end of Scenario 4, 32 (62.75%) participants were rated as “successful” and 19 (37.25%) were rated as “unsuccessful”. More importantly, 12 (42.86%) of the baseline “unsuccessful” participants successfully achieved the Scenario 4 goal, while only 3 (5.88%) of those rated as “successful” at baseline failed to do so. The proportion of those improving by Scenario 4 was significant, χ^2^ (1, N = 51) = 10.51, *p* < 0.0012. Similar to the findings of change across time on average IMPACT scores, those rated as “unsuccessful” at Scenario 1 are more likely to change success status categories by Scenario 4.

### Can verbal and nonverbal data be used to predict conversational success?

Video and audio feature sets were computed for 204 simulations (4 different scenarios per respondent, 51 respondents in total). Out of 204 simulations, 130 had a positive outcome (‘pass’, ~ 64%). A Support Vector Machine (SVM) with a linear kernel was chosen as a classifier and all results were computed using the leave-one-out cross-validation methodology. We tested several classifiers using a combination of audio and video features, sometimes limiting this to only a subset of the features. As seen from the ROC curves (see Fig. 2), the audio-only classifier gives very similar accuracy to that built on the full feature set. In order to decrease computational complexity and remove any possible security and privacy concerns associated with video streams^42^, we tried to find a minimum set of audio features so that the classifier's accuracy would be very close to that trained on a full feature set.

As seen in Fig. 2, area under the curve (AUC) for the classifier trained on top selected audio features is ~ 0.72 with an accuracy of 68%, which is very similar to both 'full' and 'audio only' classifiers. This is a promising result given that we only consider a few audio features averaged over the entire duration of the interaction and without any information on the dynamics of the conversation. To put this in perspective, an accuracy of 77% percent was achieved using video-audio non-linguistic features that included turn taking information^43^^,^^44^. Besides, this feature selection reduced the computation time needed for data preparation by ~ 85%, while the accuracy and AUC of such a classifier has a difference of only 1–2%. Note also that this feature set does not include linguistic information that may be concerning from a privacy perspective. Figure 2 also shows approximate time needed for data processing in all three cases: the full feature set, video only, and audio only.

## Discussion

The results described in the previous section highlight some important aspects of interacting with avatars that can help measure, predict, and improve an individual’s interpersonal effectiveness.

Results revealed that the interaction between the participants and the avatar produced results that we would have expected when individuals interact with each other in the real world. We attribute this similarity in results to the stimulus properties of the avatar and the scenarios. In order to allow individuals to interact with an avatar in a manner that they would have done with another person in real life, the appearance, verbal and non-verbal responses of the avatars needed to have believability and not detract from the experience. We believe that the VR software and AI algorithms (see Methods) allowed an inhabiter to effectively facilitate a natural human-avatar interaction. The results also indicate that the Scenarios which set the context of the conversation had properties that allowed the participants to interact with the avatar as they would with an individual in the real-world. Both of these lead us to believe that the presented framework reinforces the fundamental concepts of Situational Plausibility and Place Illusion^45^ required to create realistic behavior in a virtual reality setting.

Our analysis has shown a correlation between interpersonal effectiveness and success in achieving a conversational outcome, while also highlighting that interpersonal effectiveness can be learned and improved with repeated interactions with an avatar. The improvement was seen among those who were rated as “unsuccessful” at the screening conversation. By the end of four scenarios, the difference between the two groups (“successful” and “unsuccessful”) after the screening conversation was not significant, suggesting that the intervention had a positive effect. This suggests that this framework can be used to screen and target training to those who will more likely benefit from repeated interactions. While these results may include practice effects, they suggest that interpersonal effectiveness can be learned and improved across various conversational contexts. To maximize generalization, this framework provides the affordance to vary the characteristics of both the avatar and the scenario given the stimulus properties described above.

Our results also confirm that this framework offers the ability to collect high fidelity data streams which can augment our understanding of interpersonal interactions. Preliminary results of processing the audio and video from the interaction between participants and the avatar show promise in being able to predict conversational success. At this stage, we trained our predictive algorithms on features that were extracted holistically for participants that were “successful” vs. those that were “unsuccessful”. These algorithms could be further refined by extracting features in specific segments of a conversation independent of whether or not that individual was “successful” or “unsuccessful” using the IMPACT score framework. Such a self-supervised learning approach will become increasingly powerful as new data collection pipelines allow thousands of conversations to be analyzed over time. We believe that such a framework can be used to provide real-time cues to individuals as a conversation in the real-world is progressing, providing them with an opportunity to alter their behavior to ensure conversational success. The applications for such frameworks are many including sales, customer service, clinical interventions, and other professional environments.

### Comparison group, validity, & familiarity

We note that the design is quasi-experimental in the sense that we did not randomly assign participants at baseline into the successful or unsuccessful groups. Rather, group assignment was based on the performance of each participant following the baseline screening scenario. These two groups were then compared after the three “treatment” scenarios to evaluate whether additional practice resulted in improvement for both groups or was constrained to only the unsuccessful group. Our results suggest the latter. We also note that regression to the mean is a difficult confound to control given the current design. That said, if regression to the mean were likely contributing factors we would likely see it in both directions. Namely, those who scored high at the baseline screening would likely score lower on subsequent scenarios. And likewise, those who scored lower at the baseline screening would likely score higher on subsequent scenarios. The fact that we only saw a change in only one of the groups suggests that regression to the mean is an unlikely explanation of the results. Moreover, the change in the lower performing group and the lack of change in the higher performing group is consistent with our a priori hypotheses and again lends less evidence to the notion that what we observed was only regression to the mean. Finally, we analyzed the data to see if familiarity may have been a contributing factor. We noticed variability in performance across participants and scenarios. If familiarity were the only explanation, then we would expect scores to consistently improve or worsen; neither of which we observed in the data.

### Other observations for future work

After participants watched a video of their interaction with the avatar and rated their own continuous impact, they were administered a post simulation survey that asked them to assess whether or not they achieved the intended conversational outcome on a scale of 1–10. This was compared to the inhabiter’s assessment of the participant’s performance using the same survey scale. We considered participants whose assessment of their own performance was within a single point of the inhabiter’s assessment of their performance to be accurate-estimators or being “self-aware” of their performance. Similarly, over-estimators, and under-estimators, were identified depending on the direction in which their assessment differed from that of the inhabiter by 2 or more points on the scale. In total, 66% of participants were successful in their interaction with the avatar across all the simulations. Of these, ~ 78.5% were accurate-estimators or under-estimators. Of the total number of over-estimators, only ~ 50% were successful in their interaction with the avatar. In other words, participants who are self-aware of their own interpersonal effectiveness were much more likely to be successful during the interpersonal interaction than their less self-aware counterparts. We think that this result calls for future research to assess the impact of self-awareness on performance and plan to conduct follow-on studies in this area.

## Supplementary Information

Supplementary Information.

## Data Availability

All processed data from this study is available for further analysis and research in the form of .csv files hosted on figshare (Nagendran, Arjun 2022). Because of the large total size of the raw simulation videos and privacy protection requirements, the experimental data used in this work may be available upon request and provision of further details to the corresponding author.

## References

[1] Dionisio JDN, WGB III, Gilbert R (2013). 3D virtual worlds and the metaverse: Current status and future possibilities. ACM Comput. Surv. (CSUR).

[2] Mathew, O. M. The concept of avatar or avatara (incarnation) in Hinduism. Ann. de Philos. et des Sci. Hum. (2005)

[3] Bailenson JN, Blascovich JJ, Bainbridge WS (2004). Avatars. Encyclopedia of Human-Computer Interaction.

[4] Nagendran, A., Pillat, R., Hughes, C. E., & Welch, G. Continuum of virtual-human space: Towards improved interaction strategies for physical-virtual avatars. In *Proceedings of the ACM SIGGRAPH International Confer- ence on Virtual Reality Continuum and Its Applications in Industry, VRCAI*, 135–142 (2012)

[5] Blascovich J, Loomis J, Beall AC, Swinth KR, Hoyt CL, Bailenson JN (2002). Immersive virtual envi- ronment technology as a methodological tool for social psychology. Psychol. Inq..

[6] Ahn, S. J., Fox, J., & Bailenson, J. N. Avatars. In *Leadership in Science and Technology: A Reference Handbook (Chap. 79)*. (SAGE, 2012)

[7] Blascovich, J. Social influence within immersive virtual environments. In *The Social Life of Avatars* (pp. 127–145). Springer, London (2002).

[8] Fox, J., Yeykelis, L., Janssen, J. H., Ahn, S. J., Segovia, K. Y., & Bailenson, J. N. A meta-analysis quantifying the effects of avatars and agents on social influence. In *Proceedings of the National Communication Association Annual Convention, NCA *(2010).

[9] Lim S, Reeves B (2010). Computer agents versus avatars: Responses to interactive game characters controlled by a computer or other player. Int. J. Hum. Comput. Stud..

[10] O’Rourke SR, Branford KR, Brooks TL, Ives LT, Nagendran A, Compton SN (2020). The emotional and behavioral impact of delivering bad news to virtual versus real standardized patients: a pilot study. Teach. Learn. Med..

[11] Berge Z (2008). Multi-user virtual environments for education and training? A critical review of Second Life. Educ. Technol..

[12] Herrington J, Reeves TC, Oliver R (2007). Immersive learning technologies: Realism and online authentic learning. J. Comput. High. Educ..

[13] Higgins D, Fribourg R, McDonnell R (2021). Remotely perceived: Investigating the influence of valence on self-perception and social experience for dyadic video-conferencing with personalized avatars. Frontiers in Virtual Reality.

[14] Bailenson JN, Yee N, Merget D, Schroeder R (2006). The effect of behavioral realism and form realism of real-time avatar faces on verbal disclosure, nonverbal disclosure, emotion recognition and copresence in dyadic interaction. Presence Teleoper. Virtual Environ..

[15] Van Ments M (1999). The Effective Use of Role-Play: Practical Techniques for Improving Learning.

[16] Lane C, Rollnick S (2007). The use of simulated patients and role-play in communication skills training: A review of the literature to August 2005. Patient Educ. Couns..

[17] Nagendran, A., Pillat, R., Hughes, C., & Welch, G. Continuum of virtual-human space: Towards improved interaction strategies for physical-virtual avatars. VRCAI '12 (2012).

[18] Nagendran A, Pillat R, Kavanaugh A, Welch G, Hughes C (2014). A unified framework for individualized avatar-based interactions. Presence Teleoper. Virtual Environ..

[19] Hughes, C. E., Nagendran, A., Dieker, L. A., Hynes, M. C., & Welch, G. F. Applications of avatar mediated interaction to teaching, training, job skills and wellness. In *Virtual Realities* 133–146 (Springer, Cham, 2015)

[20] Lieberman MD (2013). Social: Why Our Brains are Wired to Connect.

[21] Brauer JR, Tittle CR (2012). Social learning theory and human reinforcement. Sociol. Spectrum.

[22] Mushtaq R, Shoib S, Shah T, Mushtaq S (2014). Relationship between loneliness, psychiatric disorders and physical health? A review on the psychological aspects of loneliness. J. Clin. Diagn. Res..

[23] Kunnanatt JT (2004). Emotional intelligence: The new science of interpersonal effectiveness. Hum. Resour. Dev. Q..

[24] Salovey P, Mayer JD (1990). Emotional intelligence. Imagination, cognition and personality.

[25] Miao C, Humphrey RH, Qian S (2018). A cross-cultural meta-analysis of how leader emotional intelligence influences subordinate task performance and organizational citizenship behavior. J. World Bus..

[26] Gardner WL, Fischer D, Hunt JGJ (2009). Emotional labor and leadership: A threat to authenticity?. The
Leadership Quarterly.

[27] Morand DA (2001). The emotional intelligence of managers: Assessing the construct validity of a nonverbal measure of “people skills”. J. Bus. Psychol..

[28] Haas, J. K. (2014). A history of the unity game engine.

[29] Autodesk, INC. Maya. Retrieved from https://www.autodesk.com/products/maya/ (2019).

[30] Rescorla, E. WebRTC security architecture. Work in Progress (2013).

[31] GoogleWebRTC, https://webrtc.org/ (2016).

[32] Baltrusaitis, T., Zadeh, A., Lim, Y. C., & Morency, L. P. Openface 2.0: Facial behavior analysis toolkit. In *2018 13th IEEE International Conference on Automatic Face & Gesture Recognition (FG 2018)* (pp. 59–66). IEEE (2018).

[33] Boersma P (2001). Praat, a system for doing phonetics by computer. Glot Int..

[34] Brijain, M., Patel, R., Kushik, M. R., & Rana, K. (2014). A survey on decision tree algorithms for classification.

[35] Zelenin, A., Kelly, B. D., & Nagendran, A. U.S. Patent No. 10,489,957. Washington, DC: U.S. Patent and Trademark Office (2019).

[36] The YouGov Panel, https://today.yougov.com/about/about-the-yougov-panel/

[37] Hume FMM. https://hume.ai/solutions/facial-expression-model (2022).

[38] Cowen AS, Keltner D (2020). What the face displays: Mapping 28 emotions conveyed by naturalistic expression. Am. Psychol..

[39] Ahn, J., Gobron, S., Silvestre, Q., & Thalmann, D. Asymmetrical facial expressions based on an advanced interpretation of two-dimensional russell’s emotional model. In *Proceedings of ENGAGE *(2010).

[40] Jadoul Y, Thompson B, De Boer B (2018). Introducing parselmouth: A python interface to praat. J. Phon..

[41] Nagendran, A. Avatars help improve soft skills. figshare. Dataset (2022). 10.6084/m9.figshare.20733127.v1.

[42] Kagan, D., Alpert, G. F., & Fire, M. Zooming into video conferencing privacy and security threats (2020). arXiv:2007.01059.

[43] Pentland A, Heibeck T (2008). Honest Signals.

[44] Byun, B., Awasthi, A., Chou, P. A., Kapoor, A., Lee, B., & Czerwinski, M. Honest signals in video conferencing. In *2011 IEEE International Conference on Multimedia and Expo* 1–6. IEEE (2011).

[45] Slater M (2009). Place illusion and plausibility can lead to realistic behaviour in immersive virtual environments. Philos. Trans. R. Soc. B Biol. Sci..

