# Supplementary material for: Avatar led interventions in the Metaverse reveal that interpersonal effectiveness can be measured, predicted, and improved
Source: Sci Rep. 2022 Dec 19;12:21892. doi: 10.1038/s41598-022-26326-4 (PMC9763494; doi:10.1038/s41598-022-26326-4)
Supplement: Supplementary file 1 — Supplementary Information. [file 41598_2022_26326_MOESM1_ESM.docx]

Appendix: Supplementary Information Document

**Table of Contents**

[1. Pilot Study](#_y02yqt3sgmim) 2

[1.1 Participants](#_smyvz1vn8n7q) 2

[1.2 Experimental Design & Protocol](#_lrqlzoh2646q) 2

[1.3 Results](#_2c0gk3hzjra7) 3

[2. Inhabiter Training & Instructions (Certification Process)](#_lgnti3ej96l2) 4

[3. Main Study (presented in the manuscript): Context and collected data streams](#_ak32pahfexxu) 4

[4. Main Study (presented in the manuscript): Feature Extraction in Video and Audio Streams from Simulations](#_2kkikrktvauh) 6

[5. Other Relevant Literature](#_hay9as417okw) 6

[References](#_6rhgbak4423g) 7

#

# Pilot Study

We first conducted a pilot study to develop and refine the software interface that allowed humans to interact with avatars naturally in an MLE. The software leverages Virtual Reality and Artificial Intelligence routines described in detail in the main manuscript. The pilot was intended to test the data collection and processing algorithms, enhance the user interface, and develop an interpersonal effectiveness measurement framework during human - avatar interaction that could be validated in the main study.

## 1.1 Participants

The pilot study was conducted at Mursion, a San Francisco based professional training company that delivers high fidelity avatar-based simulations across multiple industries. An e-mail was sent to all employees at Mursion describing the study and asking for volunteers to participate. Care was taken to ensure that employees understood that participation was voluntary and that not participating would in any way impact their employment status at Mursion. Fifty-nine (n=59) out of 75 employees agreed to participate in the pilot project. There were 32 female participants and 27 male participants from various departments of the company, and at various career levels within the organization, providing a reasonably representative sample of the population of employees at Mursion. Participants were provided detailed information about the purpose of the study beforehand and provided verbal informed consent prior to participating. Consent was recorded at runtime through the VR software interface. All participants were allowed to participate in the study independent of whether they were willing to provide the video recording consent, which contained data needed for analysis.

## 1.2 Experimental Design & Protocol

For the pilot study, a single topic of conversation was chosen for all participants. Participants were emailed a link to an introductory video that explained the concept of interpersonal effectiveness, and asked to utilize this framework during their interaction with the avatar. A link to the narrative of the video can be found [here](https://web.mursion.com/references/InterpersonalEffectiveness.pdf). The chosen topic of conversation (also referred to interchangeably as Scenario in the rest of the manuscript) involved addressing a communication style difference between the participants and the avatar. The description of this Scenario was available for all participants to read prior to their interaction in the MLE and can be found [here](https://web.mursion.com/references/Leadership.pdf).

In the software, participants first interacted with a host avatar so they could familiarize themselves with the interface and also ask any questions they may have about the social interaction that was about to occur. The host avatar also helped troubleshoot any issues such as sound, rendering, or network latency so the data from the actual interaction was valid and usable for analysis. Following the initial interaction with the host, participants proceeded to interact with a different avatar that represented the inhabiter in the specific Scenario. Artificial Intelligence technologies, including voice-morphing were used so that a single inhabiter could play the role of both the host avatar as well as the avatar used for the social interaction scenario in the software. The description of these technologies is described in detail in previous literature (Nagendran et al., 2012; Zelenin, A., Kelly, B. D., & Nagendran, A., 2019). Four inhabiters controlled the authoritative instance and avatars that all participants interacted with during the pilot study.

Post Interaction Questionnaire

Following the interaction, we presented several questions to the participants. The goal of these questions was to help inform a better design where we could truly measure the interpersonal performance of individuals interacting with avatars in metaversal learning environments. The questions were mostly open-ended in nature but a few contained multiple choices. The full questionnaire can be found [here](https://web.mursion.com/references/MiScoreSurvey.pdf).

## 1.3 Results

The data collected during the pilot study was analyzed to help inform a more comprehensive follow-on study. Since all data from the interactions was recorded, we benefited from an extended debrief with the inhabiters, analyzing the recorded data, and watching all the videos of the interactions. Three independent individuals were asked to watch the videos and debrief with the inhabiters while recording their observations. In addition, we correlated timestamped logs of the avatar actions with the inhabiter’s IMPACT data. Our findings are summarized below:

Measurement and Interaction Framework: From the recorded observations and the correlation analysis we learned that the body language and facial expressions of the avatar, controlled by the inhabiter via a joystick, were not aligned with the IMPACT data and events of interest data that were recorded by the inhabiter via the keyboard interface. In addition, the cognitive load on the inhabiter to use the joystick and the keyboard interfaces simultaneously was high. We overcame these limitations by using a novel audio-based AI algorithm that reduced the cognitive load of inhabiters (described in the manuscript).

Post-Interaction Questionnaire: Results of the survey revealed that more than 95% of the participants were willing to watch a video recording of their own performance, in order to help them improve their interpersonal effectiveness. In addition, participants showed a clear preference for an absolute measurement scale to indicate their interpersonal effectiveness during the interaction.

Open-Ended Findings: The post survey questionnaires also included open ended responses to help us develop a better interaction experience in the simulation. There were three major themes in the responses, illustrated by some select comments:

(i) Participants wanted to view moments in the interaction where they did something good or something bad.

“The feature of tagging moments where I initiated and responded in savvy ways with the avatar and moments where I missed her cues or went in the wrong direction would be really helpful.”

“I'd suggest providing participants with a number of "suggested markers" i.e. "Good moment - empathy", "good moment - listening", "bad moment - prescribing" etc. It would help with consistency and data metrics.”

(ii) Participants wanted to understand whether or not they improved in achieving the outcome of the conversation on a scale that was not just binary.

“No/Yes needs options in between--particularly for those who were "getting there" to feel some success and better acknowledge what did and did not go well.”

“I like the idea of someone's score being tracked against their own performances so they can clearly see improvement that has been made.”

(iii) Participants were keen on knowing how they compared to other participants who also had the same conversation.

“I think the score without detail (even if it's just how it measures up against your peers or folks doing similar scenarios) would be great”

“I could see the comparison scores being helpful within an organization, IF it is positioned as a way to gain overall relational improvement aligned with the company's culture, mission and values.”

Guided by the above findings, we proceeded to refine the software interface and algorithms, so we could conduct a comprehensive study to test our hypotheses. This forms the basis for our main manuscript.

# Inhabiter Training & Instructions (Certification Process)

The training and certification protocols described here were followed for both the pilot and the main study. Inhabiters were asked to continuously rate the learner’s performance using the guidelines below:

- If you think that the learner is saying or doing something positive **w.r.t the scenario outcome**, use the “Up arrow” to rate the learner as “Positive.”
- If you think that the learner is saying or doing something negative **w.r.t. the scenario outcome**, use the “Down arrow” to rate the learner as “Negative.”
- If you are unsure of the learner’s performance or if the learner’s impact is neither positive or negative **w.r.t. the scenario outcome**, use the “Space Bar” to rate the learner as “Neutral”

After a few such interactions, the inhabiter training team reviewed videos and data with the inhabiters, giving them feedback on using the rating interface, and seeking their feedback on how to better design the user interface. After review of several videos and the associated inhabiter data to ensure that the valence was consistent with the performance of the participant with respect to the scenario outcome, the inhabiter was considered “certified” to deliver the simulations. This process also led to improvements that have been described in the manuscript in the Rating Interface section.

# Main Study (presented in the manuscript): Context and collected data streams

Figure 1 is a schematic illustration of the architecture between the software instances used to instantiate the virtual immersive learning environment. All participants (non-authoritative instances) were located in Portland, Oregon while the inhabiter (authoritative instance) was located in Raleigh, North Carolina.


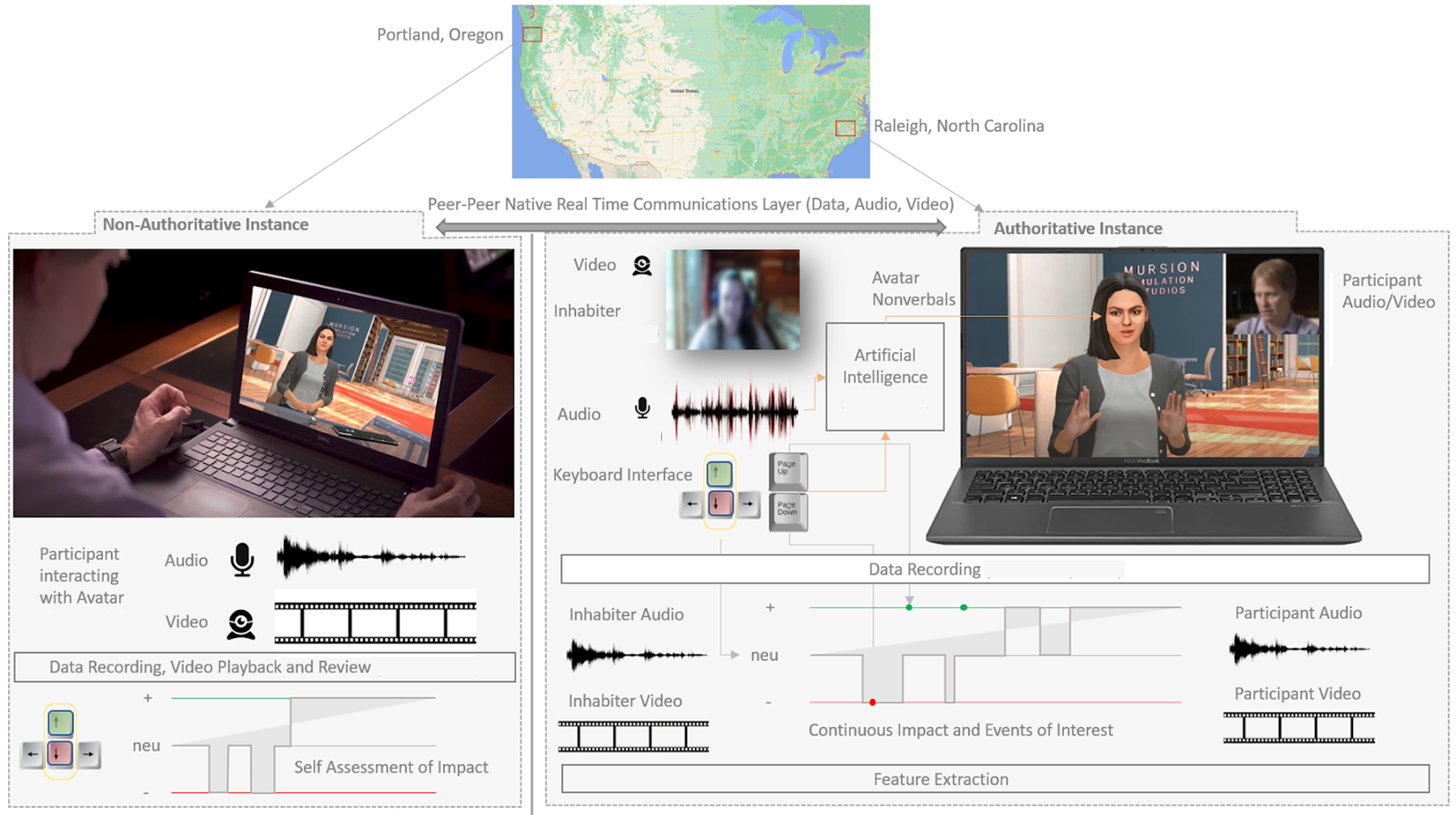


Supplementary Figure 1: Schematic showing the overall architecture of ELAINE and the data streams produced and collected by each software instance. Shown in brackets (Section) are the relevant sections of the manuscript that contain more details about the relevant architectural components. Map source: Google. (n.d.). [Google Maps view of United States of America]. Retrieved May 5, 2022, https://goo.gl/maps/kLBmqgKHDLBbK7Fw7

| **Data Stream** | **Description / Source** | **Sampling / [Resampling]** |
| --- | --- | --- |
| Participant Audio (Automated Analysis) | Spoken dialogue from the participant using a Jabra 2400 Biz Duo II noise-canceling mic and headset | 48 KHz |
| Participant Video (Automated Analysis) | Video feed of the participant from a 1080p NexiGo N60 USB Camera | 29.79 Hz |
| Inhabiter Audio (Automated Analysis) | Spoken dialogue from the inhabiter using a Jabra 2400 Biz Duo II noise-canceling mic and headset | 48 KHz |
| Inhabiter Video(Automated Analysis) | Video feed of the inhabiter from a Logitech 720p USB webcam | 15 Hz |
| The following data streams were originally collected via an event-based system but resampled to match the render frame rate of 30 frames per second for analysis: | | |
| IMPACT (Primary Outcomes) | Inhabiter’s assessment of the impact that the participant was having on their avatar | [30 Hz] |
| Events of Interest (Rating Interface) | Inhabiter’s indication of a discrete event of interest that occurred during the interaction, | [30 Hz] |

Supplementary Table 1: Details of the various data streams collected during the human-avatar interaction in the ELAINE framework.

# Main Study (presented in the manuscript): Feature Extraction in Video and Audio Streams from Simulations

Since the framework presented here provides us with a rich stream of continuous data, we analyzed these streams to discover any correlations between the underlying data and the final outcome of the interaction between the participants and the avatar. We developed a web-based interface (Figure 2) to verify the validity and integrity of all the collected data streams, allowing us to alter the thresholds used in the algorithms for voice-activity-detection, identify synchronization issues, and remove interactions that contained missing data from the analysis. Our goal was to build a machine learning classifier capable of predicting whether participants were successful or unsuccessful in their interaction with the avatar based on the data gathered during these conversational scenarios. Two primary sets of features were extracted from the collected data - video features and audio features. See Section on Automated Analysis in the main manuscript for relevant results.


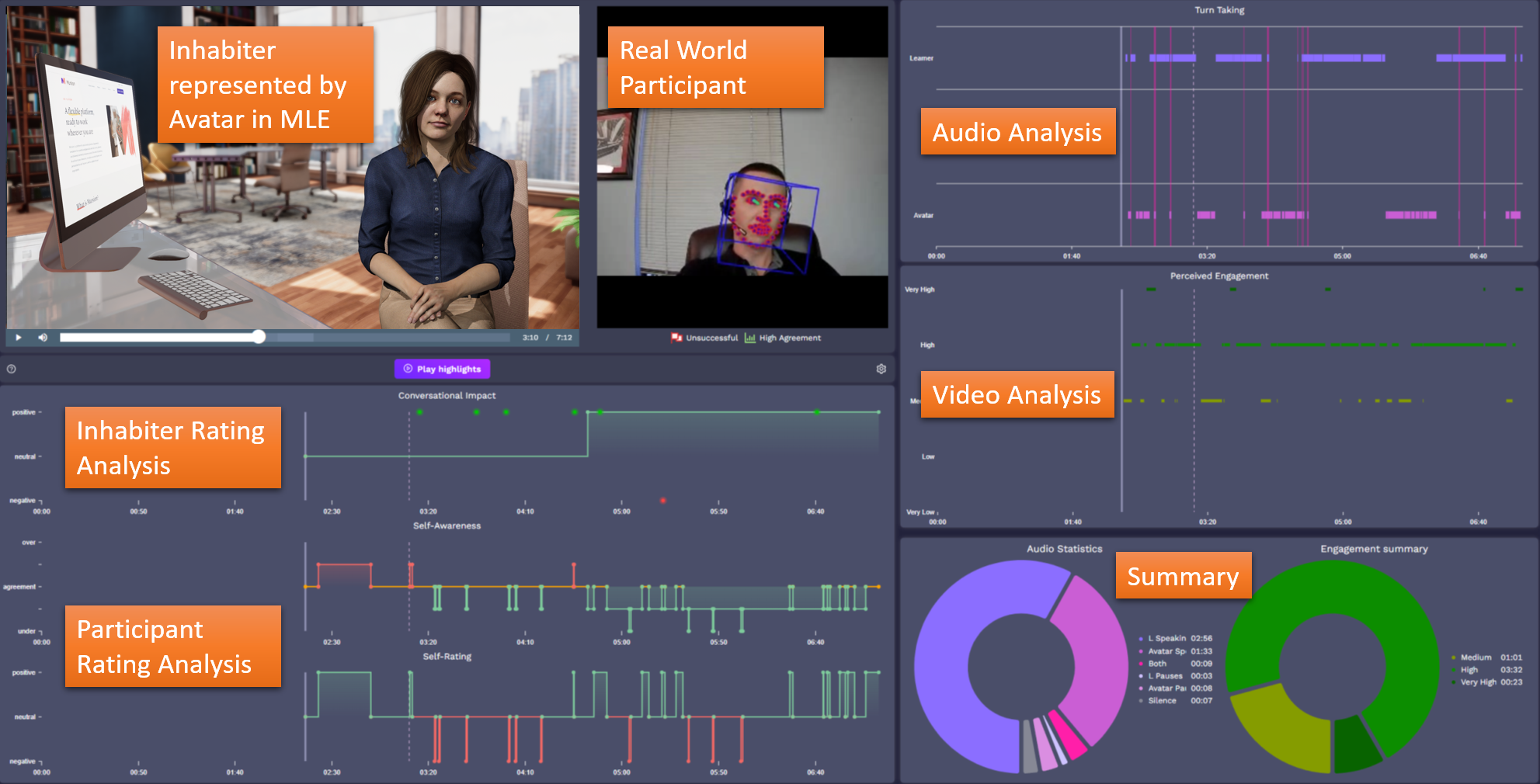
 Supplementary Figure 2: A web-based interface was developed to perform synchronized playback of the recorded data and verify the integrity and validity of all the collected data streams from the 204 simulations, prior to training the machine learning models.

1. **Other Relevant Literature**

The use of avatars in a virtual environment presents new opportunities for neuroscientists, psychiatrists and clinical psychologists to study and manipulate elements of the treatment and research settings heretofore difficult or impossible to adequately control. Avatars can elicit paranoid thoughts in patients with psychotic disorders and healthy individuals (Veling, W., Moritz, S., & van der Gaag, M. (2014)). The ability of the researcher to control voice intonation, verbal and proxemic reactions to a study participant can likely be used to describe the environmental and interpersonal features of an interaction that precipitate or mitigate psychotic responses. This presents an opportunity for patients to discriminate against safe situations and develop strategies for better reality testing. The degree of standardization of the environment and the stimulus (person) possible using VR far exceeds anything presently available.

The treatment of anxiety disorders involves exposure to stimuli that elicit fearful response so that habituation can occur (Park, M. J., Kim, D. J., Lee, U., Na, E. J., & Jeon, H. J. (2019)) . For this reason, there is a longstanding interest in the application of VR treatments to anxiety Wiederhold, B. K., & Wiederhold, M. D. (2005). In vivo exposure has long been accepted as somewhat more effective than imaginal exposure of fear inducing stimuli. However, such factors as cost, time, and safety favor imaginal exposure. Using VR allows re-creation of stimuli ranging from innocuous to highly fear evoking. The use of stimuli that closely represent the natural environment would be predicted to produce better treatment outcomes with the situation with advantages of using imaginal strategies.

# References

1. Veling, W., Moritz, S., & van der Gaag, M. (2014). Brave new worlds-review and update on virtual reality assessment and treatment in psychosis. Schizophrenia bulletin, 40(6), 1194-1197. <https://doi.org/10.1093/schbul/sbu125>
2. Park, M. J., Kim, D. J., Lee, U., Na, E. J., & Jeon, H. J. (2019). A literature overview of virtual reality (vr) in treatment of psychiatric disorders: Recent advances and limitations. Frontiers in psychiatry, 10, 505-505. https://doi.org/10.3389/fpsyt.2019.00505
3. Wiederhold, B. K., & Wiederhold, M. D. (2005). Virtual reality therapy for anxiety disorders: Advances in evaluation and treatment. American Psychological Association. https://doi.org/10.1037/10858-000
